# The Gliadin Hydrolysis Capacity of *B. longum*, *L. acidophilus*, and *L. plantarum* and Their Protective Effects on Caco-2 Cells against Gliadin-Induced Inflammatory Responses

**DOI:** 10.3390/nu15122769

**Published:** 2023-06-16

**Authors:** Najmeh Ramedani, Aurelio Seidita, Nastaran Asri, Masoumeh Azimirad, Abbas Yadegar, Somayeh Jahani-Sherafat, Anousheh Sharifan, Pasquale Mansueto, Antonio Carroccio, Mohammad Rostami-Nejad

**Affiliations:** 1 Department of Food Science and Technology, Science and Research Branch, Islamic Azad University, Tehran 9311634719, Iran; nj.ramedani@yahoo.com (N.R.); a-sharifan@srbiau.ac.ir (A.S.); 2 Department of Health Promotion Sciences, Maternal and Infant Care, Internal Medicine and Medical Specialties (PROMISE), University of Palermo, 90127 Palermo, Italy; aurelio.seidita@unipa.it (A.S.); pasquale.mansueto@unipa.it (P.M.); antonio.carroccio@unipa.it (A.C.); 3 Gastroenterology and Liver Diseases Research Center, Research Institute for Gastroenterology and Liver Diseases, Shahid Beheshti University of Medical Sciences, Tehran 1985717411, Iran; nastaran.asri26@gmail.com; 4 Foodborne and Waterborne Diseases Research Center, Research Institute for Gastroenterology and Liver Diseases, Shahid Beheshti University of Medical Sciences, Tehran 1985717411, Iran; rad.masy@gmail.com (M.A.); babak_y1983@yahoo.com (A.Y.); 5 Laser Application in Medical Sciences Research Center, Shahid Beheshti University of Medical Sciences, Tehran 1416634793, Iran; jahani_somayeh@yahoo.com; 6 Celiac Disease and Gluten Related Disorders Research Center, Research Institute for Gastroenterology and Liver Diseases, Shahid Beheshti University of Medical Sciences, Tehran 1985717411, Iran

**Keywords:** gliadin, non-celiac wheat sensitivity, probiotics, Caco-2 cells

## Abstract

Background: Non-celiac wheat sensitivity (NCWS) is a poorly understood gluten-related disorder (GRD) and its prominent symptoms can be ameliorated by gluten avoidance. This study aimed to determine the effectiveness of a probiotic mixture in hydrolyzing gliadin peptides (toxic components of gluten) and suppressing gliadin-induced inflammatory responses in Caco-2 cells. Methods: Wheat dough was fermented with a probiotic mix for 0, 2, 4, and 6 h. The effect of the probiotic mix on gliadin degradation was monitored by SDS-PAGE. The expression levels of IL-6, IL-17A, INF-γ, IL-10, and TGF-β were evaluated using ELISA and qRT-PCR methods. Results: According to our findings, fermenting wheat dough with a mix of *B. longum*, *L. acidophilus*, and *L. plantarum* for 6 h was effective in gliadin degradation. This process also reduced levels of IL-6 (*p* = 0.004), IL-17A (*p* = 0.004), and IFN-γ (*p* = 0.01) mRNA, as well as decreased IL-6 (*p* = 0.006) and IFN-γ (*p* = 0.0009) protein secretion. 4 h fermentation led to a significant decrease in IL-17A (*p* = 0.001) and IFN-γ (*p* = 0.003) mRNA, as well as reduced levels of IL-6 (*p* = 0.002) and IFN-γ (*p* < 0.0001) protein secretion. This process was also observed to increase the expression levels of IL-10 (*p* < 0.0001) and TGF-β (*p* < 0.0001) mRNA. Conclusions: 4 h fermentation of wheat flour with the proposed probiotic mix might be a good strategy to develop an affordable gluten-free wheat dough for NCWS and probably other GRD patients.

## 1. Introduction

Gluten-related disorders (GRDs), an “umbrella” definition encompassing several diseases, including non-celiac wheat sensitivity (NCWS), are becoming increasingly frequent. Gluten is a protein present in wheat, barley, rye, and occasionally in other grains, that is responsible for the elasticity and pliability of dough, making it an essential ingredient in various baked goods, such as bread, pasta, and pastries. Consumption of this protein by GRD patients can lead to pathological conditions and should thus be avoided by them [1]. Contributory factors to the growing prevalence of GRDs [2] are agricultural mechanization and the excessive reliance on industrial pesticides, which have stimulated the development of novel varieties of wheat, characterized by a higher content of toxic peptides of gluten. Furthermore, the current state of bread and bakery production methods has resulted in a reduction in fermentation time for dough and the use of chemical agents or baker’s yeast. This, in turn, has been observed to result in a significant rise in the [amount of] gluten present in the end products, consequently exacerbating the clinical state of GRD patients [2]. Therefore, there has been a notable increase in the demand for gluten-free bakery products, particularly among groups who suffer from this kind of disorders [3,4,5,6,7].

NCWS, also commonly referred to as non-celiac gluten sensitivity (NCGS), gluten hypersensitivity, or wheat intolerance syndrome, is a poorly understood GRD that has a self-reported prevalence in the general population ranging from 0.6% to 13% [3,4,5,6,7]. NCWS appears to be triggered by activation of the innate and/or adaptive immune responses and its main pathological characteristic might be an alteration in intestinal permeability [8,9,10,11,12]. Currently, NCWS diagnosis entails ruling out other GRDs, such as celiac disease (CD) and IgE-mediated wheat allergy, as well as organic diseases in subjects on a gluten-containing diet. This involves observing symptom improvement after gluten elimination followed by relapse during a double-blind placebo-controlled gluten/wheat challenge [5,13,14,15,16,17]. Unfortunately, unlike CD, histology on duodenal biopsy specimens does not help in NCWS diagnosis. There are a few reports regarding the histopathology of NCWS, but a maintained villous architecture, a preserved villus/crypt ratio, a normal or mildly increased level of intraepithelial lymphocytes, and an increased number of eosinophils and mast cells in the duodenal mucosa are among the histopathological findings reported to date [6,8,18,19,20,21,22,23,24,25,26]. NCWS presents with an extensive range of symptoms, both intestinal and extraintestinal. These vary from IBS- and dyspepsia-like symptoms to neuropsychiatric ones, anemia, and other disorders that impact multiple organ systems [11,27,28,29,30,31]. 

Adherence to a gluten-free diet (GFD) can significantly alleviate or eliminate the prominent symptoms experienced by individuals with NCWS, although some individuals may require additional dietary interventions, such as a low FODMAP diet [32,33,34,35,36,37,38,39]. Whereas gluten has been considered as the main culprit for symptom onset in GRD patients, other components of wheat, such as amylase trypsin inhibitors (ATIs), wheat germ agglutinins (WGAs), and fermentable oligo-, di-, monosaccharides, and polyols (FODMAPs), are also reported to contribute to the development of NCWS [5,31,32,33].

However, a GFD, similar to most other elimination diets, can be accompanied by potential nutritional deficiencies due to the elimination of important food sources, such as wheat, and needs to be supervised by a qualified health care provider [40]. Moreover, the unavailability of gluten-free foods, their high cost, and problems with eating out or travelling are known as burdens faced by subjects on a GFD and reduce diet adherence [41].

Therefore, food-processing researchers are looking to develop and market high-quality gluten-free products with an affordable price [42]. One proposal is a return to sourdough fermentation, an ancient bread-making technique using lactic acid bacteria (LAB) and natural yeast populations to transform cereal flour into end products, which are tastier and easier to digest [34]. Various studies have confirmed its positive effects on immune response and NCWS symptoms [43].

According to previous findings, there are probiotic strains that have the ability to degrade immunodominant gliadin peptides and can potentially reduce their toxicity [44,45,46]. *Bifidobacteria* and *lactobacilli* are two widely used probiotics with gluten protein hydrolyzing capability [47]. Use of these probiotic strains during fermentation might be beneficial in producing a less immunogenic wheat flour and reintroducing one of the most important components of the Mediterranean diet to NCWS patients’ nutrition regimen. The Caco-2 enterocyte cell line is considered to be a validated in vitro model to evaluate the cytotoxic effect of gliadin-derived peptides. It has been extensively utilized in previous GRD-related studies to help determine the pathogenesis of these diseases [48,49,50].

The current study therefore aimed to investigate the enzymatic hydrolysis activity of *Bifidobacterium Longum*, *Lactobacillus acidophilus*, and *Lactiplantibacillus plantarum* on gliadin peptides of wheat flour and their potential effect on gliadin-related inflammatory responses in the Caco-2 cell line. 

## 2. Materials and Methods

Investigations were carried out at the Research Institute for Gastroenterology and Liver Diseases (RIGLD), Shahid Beheshti University of Medical Sciences, Tehran, Iran. Data validation was carried out at the Department of Health Promotion Sciences, Maternal and Infant Care, Internal Medicine and Medical Specialties (PROMISE), University of Palermo, Palermo, Italy.

### 2.1. Probiotic Preparations

All three lyophilized probiotics, including *Bifidobacterium longum* (ATCC 15708), *Lactobacillus acidophilus* (ATCC 4356) and *Lactiplantibacillus plantarum* (ATCC 8014), were purchased from Takgene Group Company (Tehran, Iran) and used for dough fermentation. The bacteria were cultured in an MRS (De Man, Rogosa and Sharpe) medium supplemented with 0.05% (*v*/*v*) L-cysteine at 37 °C for 24–48 h in anaerobic conditions. The strains were kept at −80 °C in MRS broth plus L-cysteine and 50% glycerol.

### 2.2. Sourdough Fermentation

The wheat grain (Mehregan cultivar) with a gluten content of 27.3% was provided by the Agricultural Research, Education, and Extension Organization (AREEO), Karaj, Iran. For the preparation of the sourdough, 80 g of wheat flour was mixed with 190 mL of tap water to obtain 270 g of sourdough. The resulting dough was then divided into two 135 g pieces. The first one was mixed with a combination of the three bacteria, at a cell concentration of ca. 10^8^ CFU per g of dough. The second was used without probiotics as a negative control. Both dough pieces were incubated at 37 °C during stirring at 200 rpm. The capacity of *B. longum*, *L. plantarum,* and *L. acidophilus* to hydrolyze gluten was evaluated at 0, 2, 4, and 6 h of incubation.

### 2.3. Gliadin Extract

For gliadin extraction, approximatley 10 g of each dough sample was incubated with 50 mM Tris-HCl buffer (pH = 8.8) for 60 min at 4 °C followed by vortexing and centrifuging at 20,000× *g* for 20 min. After removing the supernatant that contained albumin and globulin, the pellet was extracted twice with Tris-HCl buffer (pH = 8.8), and the supernatant was discarded. The pellet was then mixed with 40 mL of 70% *v*/*v* ethanol at 25 °C for 2 h. The supernatant containing gliadin was stored at −80 °C. The concentration of the extracted gliadin was determined using a bicinchoninic acid (BCA) protein assay kit (DNAbiotech, Tehran, Iran). 

### 2.4. SDS-PAGE Electrophoresis of Gliadins

Gliadin degradation after 0, 2, 4, and 6 h of incubation with a mix of *B. longum*, *L. acidophilous*, and *L. plantarum* was assessed by sodium dodecyl-sulfate polyacrylamide gel electrophoresis (SDS-PAGE). A mini SDS-PAGE (Bio-Rad Laboratories GmbH, Munich, Germany) was used, separation was performed using the classical Laemmli method [51], and the bands were stained with Coomassie Blue.

### 2.5. In Vitro Digestion of Gliadins

A pepsin/trypsin (PT) digest of extracted gliadins (to mimic normal intestinal degradation) was prepared to be exposed to the human colon carcinoma Caco-2 cell line, as follows: 120 mg of extracted gliadin was dissolved in 20 mL of sodium acetate buffer (50 mM, pH 4). The sample was incubated at 37 °C, with agitation for 2 h after the addition of pepsin (3200 U/mg, P-6887, Sigma-Aldrich, St. Louis, MO, USA) to the mixture. After incubation, 142 mg of sodium phosphate was added, and the pH was brought to 7.0 using a 1 M NaOH solution. The mixture was treated with trypsin (2500 U/mg, T-7418, Sigma-Aldrich, St. Louis, MO, USA) and incubated at 37 °C with agitation for another 2 h. The obtained solution was heated at a temperature greater than 95 °C for 10 min to stop the enzymatic reaction. Finally, the digested solution was lyophilized and stored at −20 °C. 

### 2.6. Cell Culture Conditions

Caco-2 cells were grown in minimum essential medium (MEM, Gibco Invitrogen, Paisley, UK) supplemented with 10% fetal bovine serum (FBS, Gibco Invitrogen), 1% non-essential amino acids (NEAA, Gibco Invitrogen), 0.1% penicillin-streptomycin (Gibco Invitrogen), sodium bicarbonate (Gibco Invitrogen), and sodium pyruvate (Sigma-Aldrich, Seelze, Germany). Cells were maintained at 37 °C in 5% CO_2_ and the culture medium was changed every 3 days until confluency reached 80%.

Untreated Caco-2 cells were used as negative controls to assess the base levels of cytokines secretion. LPS-treated Caco-2 cells were used as positive control to assess the levels of cytokines secretion under inflammatory conditions.

### 2.7. Evaluating B. longum, L. acidophilous, and L. plantarum Effects on the Inflammatory Effects of Gliadin Peptides in Intestinal Epithelial (Caco-2) Cells

24 h after seeding, cells were incubated with 1 mg/mL of PT-digest of gliadins (extracted from 0, 2, 4, and 6 h probiotic fermented and unfermented doughs) [52], 1 μg/mL of LPS (Lipopolysaccharides, as a pro-inflammatory response triggering control) [53], and *B. longum*, *L. acidophilous*, and *L. plantarum* (alone in a mix) (Multiplicity of Infection (MOI) = 100), and the incubation was continued for 24 h. Following incubation, cells and supernatants were collected and analyzed for the production of pro- and anti-inflammatory cytokines.

### 2.8. Sandwich Enzyme-Linked Immunosorbent Assay (ELISA)

To evaluate IL-6 and INF-γ production as proinflammatory cytokines, the supernatant was obtained, and protein concentrations were determined using the enzyme-linked immunosorbent assay (ELISA) technique (Karmania Pars Gene, Rafsanjan, Iran) according to the manufacturer’s instructions. Triplicate results were read at 450 nm by an ELISA reader (TECAN, Salzburg, Austria).

### 2.9. RNA Extraction and Quantitative Real-Time PCR (qRT-PCR)

A YTA Total RNA Purification mini kit for the Blood/Cultured Cell/Tissue kit (Yekta Tajhiz Azma, Tehran, Iran) was used to extract the total RNA from Caco-2 cells. The NanoDrop-1000 spectrophotometer (Nano Drop Technologies, Wilmington, DE, USA) was used to measure the concentration and purity of the extracted RNA. Reverse transcription was performed using the 2 step 2X qRT-PCR Premix (Taq) kit (BioFact™, Daejeon, Republic of Korea), and to investigate the expression levels of IL-6, INF-γ, IL-17, IL-10, and TGF-β as pro- and anti-inflammatory cytokines, a SYBR Green-based quantitative real-time PCR assay was performed using the Rotor-Gene Q MDx. Gene Runner software version 6.0 was used to design the primer sequences and their specificity was checked by NCBI Primer-BLAST and performing PCR experiments followed by 1.5% agarose gel electrophoresis. Glyceraldehyde-3-phosphate dehydrogenase (GAPDH) was used as the reference housekeeping gene and relative quantitation (RQ) for each gene expression calculated by the 2^−ΔΔCt^ method.

### 2.10. Statistical Analysis

Data were analyzed using Prism 6.04 for Windows software (GraphPad, La Jolla, CA, USA). Data were expressed as mean ± standard deviation (SD), and one-way ANOVA were used to evaluate differences in means between groups. *p* values below 0.05 were deemed statistically significant.

### 2.11. Ethical Approval

This article does not contain any studies with human or animal subjects and was approved by the ethics committee of the Research Institute for Gastroenterology and Liver Diseases Shahid Beheshti University of Medical Sciences, Tehran, Iran (IR.SBMU.RIGLD.REC.1397.010).

## 3. Results

### 3.1. Gliadin Hydrolysis by Selected Probiotics

**Figure 1:**
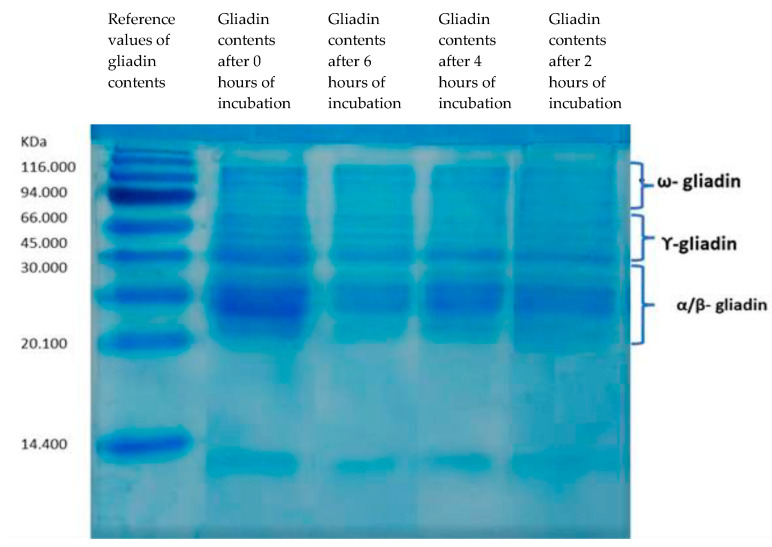
SDS-PAGE. Degradation of gliadin by a mix of *B. longum*, *L. acidophilous*, and *L. plantarum* after 0, 6, 4, and 2 h of incubation.

According to the SDS-PAGE profiles of the gliadin polypeptides, a 6 h fermentation of dough with a mix of *B. longum*, *L. acidophilous*, and *L. plantarum* showed the highest gliadin hydrolysis capacity (Figure 1). 

### 3.2. IL-6 and INF-γ Protein Expression

**Figure 2:**
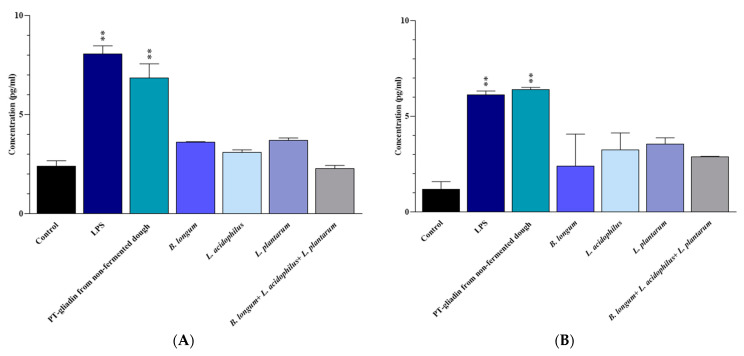
(**A**) IL-6 and (**B**) IFN-γ levels in the culture medium of Caco-2 cells after 24 h of treatment. Results are expressed in pg/mL as the mean ± SD. Control: Untreated Caco-2 cells. LPS-treated Caco-2 cells were used as positive control. **: *p* ≤ 0.01.

Compared to the untreated Caco-2 cells (negative controls), the Caco-2 cells that were treated for 24 h with LPS (positive controls) and non-fermented-dough-extracted PT-gliadin exhibited significantly higher levels of IL-6 (*p* < 0.0001 in both cases) and IFN-γ (*p* = 0.003 and 0.002, respectively). No significant difference in the secretion of the two pro-inflammatory cytokines was observed between LPS and non-fermented dough-extracted PT-gliadin treated Caco-2 cells, as well as between untreated Caco-2 cells and those treated with *B. longum*, *L. acidophilus*, and *L. plantarum*, alone or in a mix (*p* > 0.05) (Figure 2).

**Figure 3:**
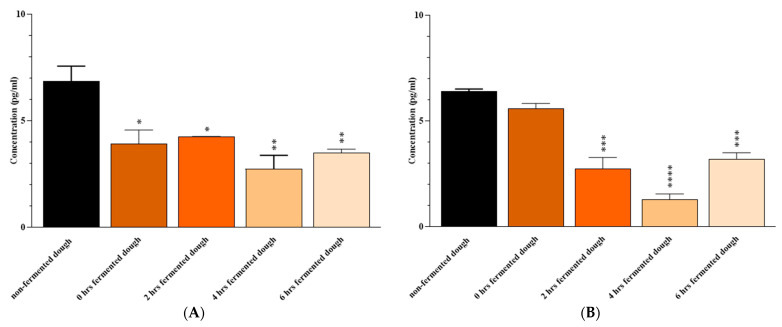
(**A**) IL-6 and (**B**) IFN-γ levels the culture medium of Caco-2 cells after 24 h of exposure to non-fermented or 0-, 2-, 4- and 6-h fermented dough. Results are expressed in pg/mL as the mean ± SD. Non-fermented dough-extracted PT-gliadin-triggered Caco-2 cells were used as positive controls. *: *p* ≤ 0.05, **: *p* ≤ 0.01, ***: *p* ≤ 0.001, ****: *p* ≤ 0.0001.

Exposure of Caco-2 cells to 2, 4, and 6 h fermented wheat dough with a mix of *B. longum*, *L. acidophilous*, and *L. plantarum* resulted in lower levels of IL-6 (*p* = 0.02, 0.002, and 0.006, respectively) and IFN-γ (*p* = 0.0005, <0.0001, and 0.0009, respectively) compared to ones from non-fermented dough-extracted PT-gliadin-triggered Caco-2 cells (which were used as positive controls). The reduction in IL-6 was also observed after 0 h of fermentation (*p* = 0.01) (Figure 3).

### 3.3. IL-6, IL-17A, IFN-γ, IL-10, and TGF-β mRNA Expression

**Figure 4:**
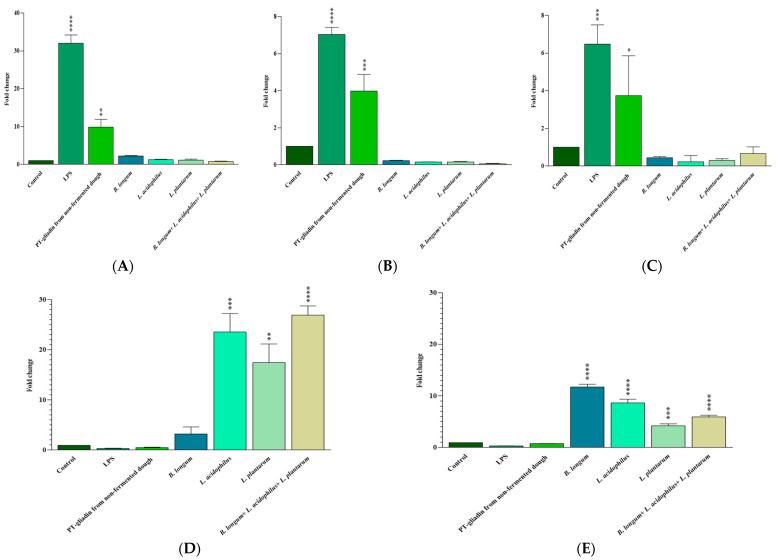
The expression of IL-6 (**A**), IL-17A (**B**), IFN-γ (**C**), IL-10 (**D**) and TGF-β (**E**) genes in treated Caco-2 cells was measured by qRT-PCR and compared to the untreated and LPS treated Caco-2 cells. Results are expressed as the mean ± SD. Control: Untreated Caco-2 cells. LPS-treated Caco-2 cells were used as positive control. *: *p* ≤ 0.05, **: *p* ≤ 0.01, ***: *p* ≤ 0.001, ****: *p* ≤ 0.0001.

To assess the probiotic effects on pro- and anti-inflammatory responses we studied IL-6, IL-17A, IFN-γ, IL-10. and TGF-β gene expression. Treatment of Caco-2 cells with LPS (positive control) and non-fermented dough-extracted PT-gliadin for 24 h resulted in a higher mRNA expression of IL-6 (*p* < 0.0001 and *p* = 0.001, respectively), IL-17A (*p* < 0.0001 and *p* = 0.0009, respectively), and IFN-γ (*p* = 0.0003 and *p* = 0.04, respectively) compared to the untreated cells (negative control). Caco-2 cell treatment with *B. longum*, *L. acidophilous*, and *L. plantarum* alone and in a mix increased IL-10 (*p* > 0.05, 0.0002, 0.001, and <0.0001, respectively) and TGF-β (*p* = 0.0005 for *L. plantarum*, *p* < 0.0001 for the others) mRNA expressions in comparison to the untreated cells (Figure 4). There was no significant difference between the probiotic-treated and untreated groups in terms of pro-inflammatory cytokine (IL-6, IL-17A, and IFN-γ) expression (*p* > 0.05). Finally, no difference was proved between LPS and non-fermented dough-extracted PT-gliadin treated Caco-2 cells both in terms of pro- and anti-inflammatory mRNA cytokines expression.

**Figure 5:**
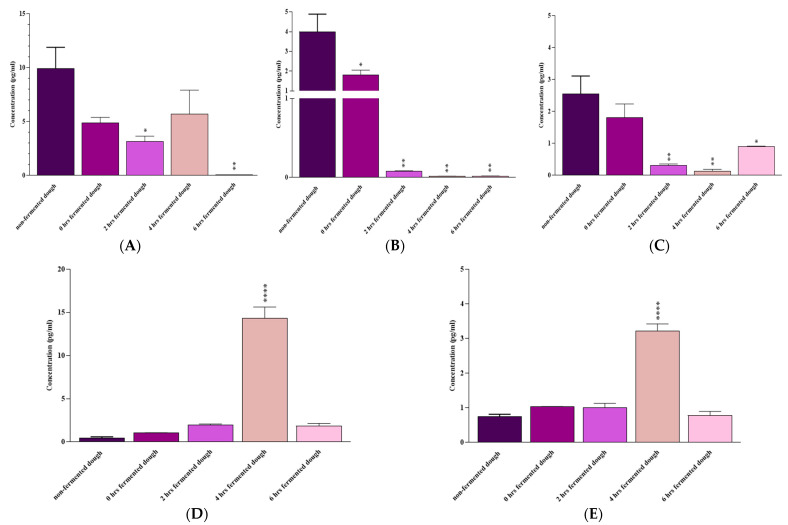
(**A**) IL-6, (**B**) IL-17A, (**C**) IFN-γ, (**D**) IL-10, and (**E**) TGF-β mRNA expression by Caco-2 cells after 24 h of exposure to non-fermented or 0, 2, 4, and 6 h fermented dough. Results are expressed as the mean ± SD. Non-fermented-dough-extracted PT-gliadin-triggered Caco-2 cells were used as positive controls. *: *p* ≤ 0.05, **: *p* ≤ 0.01, ****: *p* ≤ 0.0001.

As shown in Figure 5, fermenting the dough with a mix of *B. longum*, *L. acidophilous*, and *L. plantarum* for 6 h significantly reduced IL-6 (*p* = 0.004), IL-17A (*p* = 0.004), and IFN-γ (*p* = 0.01) mRNA expressions in comparison with non-fermented dough-extracted PT-gliadin-incubated Caco-2 cells. IL-10 and TGF-β levels were slightly higher following 6 h of fermentation with the probiotic mix; however, the changes were not statistically significant (*p* > 0.05).

A 4 h fermentation with these probiotics considerably decreased IL-17A (*p* = 0.001) and IFN-γ (*p* = 0.003), increased IL-10 (*p* < 0.0001) and TGF-β (*p* < 0.0001), and reduced (not statistically significant) IL-6 (*p* > 0.05) mRNA levels compared to the non-fermented dough-extracted PT-gliadin-incubated Caco-2 cells.

Finally, 2 h of fermentation with probiotics was also effective in reducing IL-6 (*p* = 0.02), IL-17A (*p* = 0.001), and IFN-γ (*p* = 0.004) gene expression, but its effect on IL-10 and TGF-β mRNA levels was not statistically significant (*p* > 0.05) compared to the non-fermented dough-extracted PT-gliadin-incubated Caco-2 cells.

Compared to non-fermented dough-extracted PT-gliadin-incubated Caco-2 cells, a 0-h fermentation with these probiotics only reduced IL-17A mRNA expression (*p* = 0.01).

## 4. Discussion

Sourdough fermentation is a traditional process for bread leavening that may markedly reduce wheat allergens and break down gluten, but it does not eliminate gluten proteins completely [43,54]. Therefore, researchers are trying to find novel biotechnological strategies based on using probiotics to make almost totally gluten-free products more accessible [55]. Probiotics have been shown to be an excellent source of endopeptidases, which can reduce gluten content in wheat foods and seem to be the strongest candidates for improving the quality of gluten-free foods [42]. In this regard, it has been reported that “ancient” wheats, which partially differ in composition from “modern” wheat species, seem to be better tolerated (lower rate of symptom onset, better palatability, and lower cost than gluten-free products) by a subset of NCWS patients [56,57]. As *Bifidobacteria* and *Lactobacilli* strains are known to be the most common probiotics used in foods and supplements, we evaluated the effect of a probiotic mix containing *B. longum*, *L. acidophilous*, and *L. plantarum* on degrading gliadin peptides of wheat flour dough and reducing their inflammatory effects in a Caco-2 cell line. Similar to previous in vitro studies, we observed the inflammatory effect of peptic–tryptic digested gliadin peptides (extracted from non-fermented dough) on the Caco-2 intestinal cells [58,59]. Our results also showed the capacity of *B. longum*, *L. acidophilous*, and *L. plantarum* to reduce pro-inflammatory and increase anti-inflammatory cytokine expression by these cells. Álvarez-Mercado et al., in a recent study, observed how *B. longum* subsp. *Infantis* reduced the secretion of pro-inflammatory cytokines by Caco-2 cells [59]. Another study, conducted by Zhao et al., reported that *B. longum* subsp. *longum K5* produced a decrease in the levels of pro-inflammatory cytokines and inflammatory mediators in RAW 264.7 and Caco-2 cells stimulated with LPS [60]. The inflammatory regulation role of *L. acidophilus* has also been observed in human intestinal epithelial cells [61] and human MKN45 cells [62]. An attenuating effect of *L. plantarum MYL26* on inflammation in Caco-2 cells was also reported by Chiu et al. [63].

Scricciolo et al. [64], in a recent randomized, double-blind, placebo-controlled monocentric study, did not observe any effectiveness of using a proline-specific endopeptidase (P1016) to improve NCWS patients’ symptoms following gluten reintroduction. On the contrary, in a study conducted by Ido et al. [65], an enzyme mix sourced from microorganisms and papaya was found to effectively reduce symptoms in NCWS patients undergoing a gluten challenge. In detail, the authors used a mixture of peptidase, semi alkaline protease, deuterolysin, and cysteine protease derived from *Aspergillus oryzae*, *Aspergillus melleus*, *Penicillium citrinum*, and *Carica papaya L.*, respectively. A group of thirty patients diagnosed with NCWS were randomly assigned to a two-week single-blind challenge with gluten plus either the enzyme mix or placebo. The patients then underwent a crossover phase after a GFD wash-out period. The authors observed a significant difference in the overall symptom score between post-washout and post-gluten challenge stages for the enzyme mixture and placebo groups (*p* < 0.05). However, there were no noticeable differences in serum levels of IL-8, TNF-α, or RANTES after the consumption of gluten, gluten with the enzyme mix, or gluten with a placebo [65].

In our study, we demonstrated the desirable effects of probiotics for producing easily available gluten-free wheat flour. The fermentation of wheat dough using a mix of *B. longum*, *L. acidophilus*, and *L. plantarum* for 6 h was found to be effective in gliadin degradation and decreased IL-6, IL-17A, and IFN-γ mRNA levels, as well as IL-6 and IFN-γ protein secretion. However, a 4 h fermentation with this mix might be the optimal protocol for making gluten-free wheat flour dough suitable for patients with GRD. This is due to the strong ability of the 4 h fermentation to decrease pro-inflammatory cytokine mRNA expression (IL-17A and IFN-γ) or protein secretion (IL-6 and IFN-γ), and at the same time, increase anti-inflammatory cytokine mRNA levels (IL-10 and TGF-β). These results agree with those of previous studies reporting protective effects of certain probiotic strains against some gliadin-mediated inflammatory responses. In a previous study, *Enterococcus mundtii QAUSD01* and *Wickerhamomyces anomalus QAUWA03* were reported to be able to degrade gliadin in various wheat varieties [66]. Probiotic VSL#3 (Ferring Pharamaceuticals, Milan, Italy), containing *Streptococcus thermophilus*, *L. plantarum*, *L. acidophilus*, *L. casei*, *L. delbrueckii* spp. *bulgaricus*, *B. breve*, *B. longum,* and *B. infantis*, has also been reported to decrease the toxicity of wheat flour following a 24 h fermentation [67]. In addition, Leszczyn’ska et al. reported that a 24 h fermentation of wheat flour with *L. plantarum*, *L. brevis*, *L. sanfranciscencis,* and bakery yeasts (*Saccharomyces cerevisiae*) lowered the allergenicity of wheat flour [68].

The optimal therapeutic approach for patients with NCWS remains a topic of ongoing debate among researchers [28,29,30,31]. This debate is attributable to the lack of a univocal understanding of the etiopathogenesis of NCWS. Research groups hold conflicting views, often citing gluten, WGAs, ATIs, or FODMAPs as the sole cause behind clinical manifestations in NCWS patients [26,29,30,31,69]. On the basis of the current understanding of this GRD, NCWS should be viewed as a complex entity, with several subtypes of patients. Multiple studies have shown that some individuals with NCWS experience symptom improvement or remission by adhering to a GFD, and their symptoms worsen during double-blind placebo-controlled (DBPC) challenges with gluten [14,29,70,71,72]. Contrary to this, some studies suggest that simply reintroducing gluten is not enough to re-trigger symptoms. Instead, it is believed that other components present in wheat could also play a role [30,31,69]. To date, no study has defined whether there is a tolerability threshold for these components of wheat. Moreover, the limited availability of comprehensive long-term research studies does not permit any definite conclusions as to whether NCWS is a persistent condition. Consequently, it remains uncertain if patients can regain wheat tolerance after years of avoiding the triggering stimuli.

Our study allowed us to identify a mix of probiotics easily available on the market, which, by digesting gliadin peptides in a relatively short time (4 h), are able to reduce the inflammatory and increase the anti-inflammatory response in an in vitro model with Caco-2 cells. Its most promising use could be as a starter to ferment the dough of wheat, creating a tolerable, palatable, easily accessible, and low-cost product, which could be potentially tolerated by a subgroup of NCWS subjects, as well as other GRD patients and at the same time prevent the onset of nutritional deficiencies.

The primary limitation of our study is its exclusive reliance on in vitro data. The potential tolerance of wheat dough fermented using our probiotic mix needs to be verified in appropriately designed in vivo studies. Another limitation is that we assessed the effectiveness of the probiotic mix solely on the Mehregan cultivar of wheat. As a result, we cannot rule out potential variations in the efficacy of the mix when applied to other types of wheat, with different percentages of gliadin peptides and compositions. To address these limitations, we have initiated plans for a multicenter study. This new study aims to test the effectiveness of the probiotic mixture in gliadin degradation and tolerance in NCWS patients using wheat dough obtained from various Italian and Iranian cultivars, including both modern and ancient varieties, and through both in vitro and in vivo assessments.

## 5. Conclusions

Adhering to a GFD can be extremely challenging, particularly due to the limited availability of gluten-free food options. Therefore, finding a probiotic mixture that can help make gluten-free wheat flour available in a short time could represent a significant advancement towards enhancing the quality of life for the countless individuals worldwide grappling with gluten-related disorders. According to our findings, a 4 h fermentation of wheat flour with a probiotic mixture containing *B. longum*, *L. acidophilus*, and *L. plantarum* (ca. 10^8^ CFU per g of dough) might be a good strategy for developing an affordable gluten-free wheat dough, which can be used to prepare different food items for these patients. However, our understanding of the exact molecular mechanisms underlying the action of these probiotics is currently limited and requires further investigation.

## Data Availability

The data presented in this study are available on request from the corresponding author.
